# Supplementary material for: Saccharomyces boulardii (CNCM I-745) improves intestinal damage in sepsis by remodeling intestinal flora structure
Source: Front Cell Infect Microbiol. 2025 Jul 31;15:1602792. doi: 10.3389/fcimb.2025.1602792 (PMC12350329; doi:10.3389/fcimb.2025.1602792)
Supplement: Supplementary file 1 [file DataSheet1.zip › summary/summary/7_Differential_analysis/1_diff_abundance/6_Genus/Group.DW4_vs_DW0_vs_CW4_vs_CW0_vs_BW4_vs_BW0_vs_AW4_vs_AW0/Group.DW4_vs_DW0_vs_CW4_vs_CW0_vs_BW4_vs_BW0_vs_AW4_vs_AW0_diff_barplot.pdf]

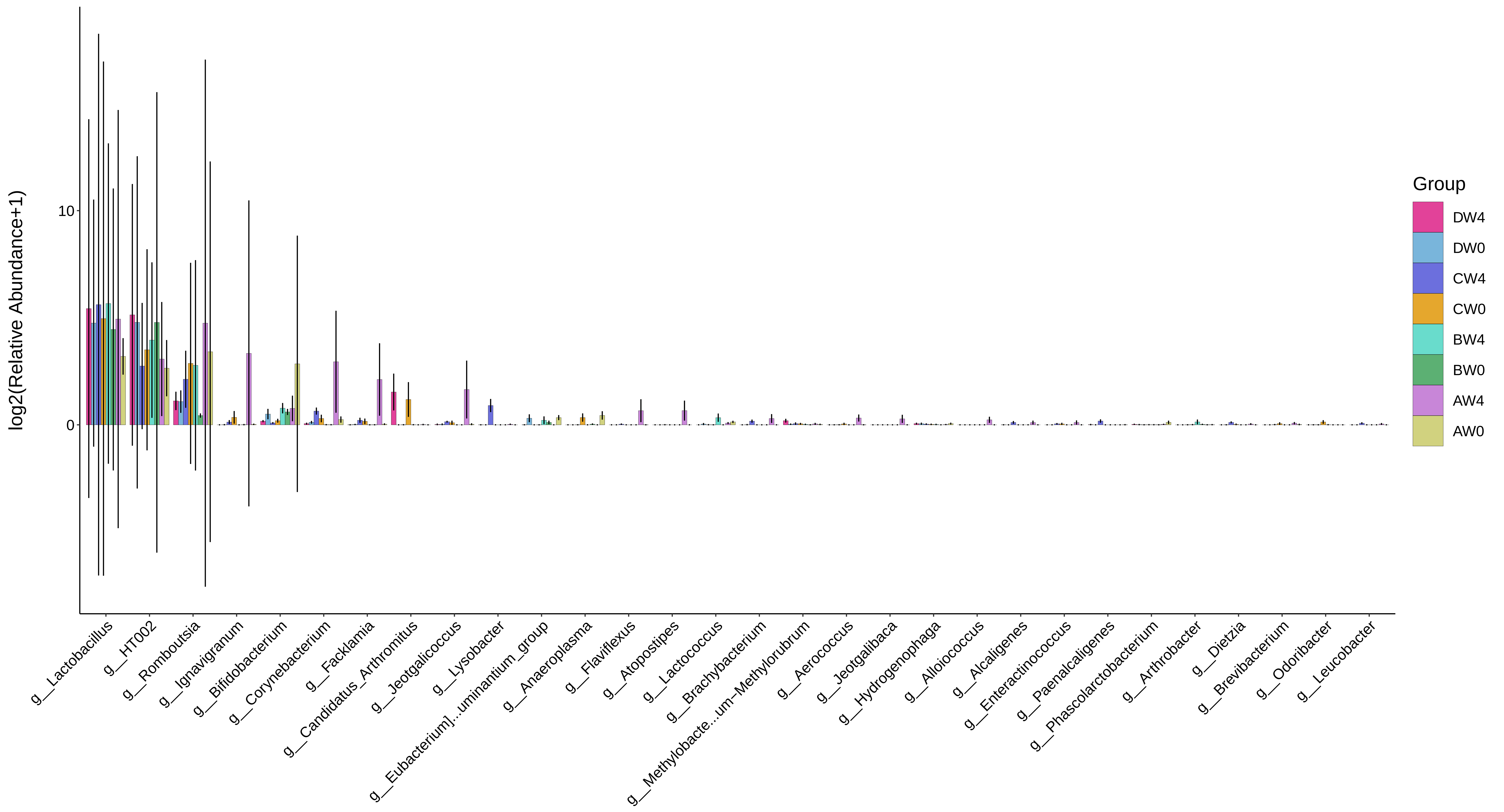

log2(Relative Abundance+1)
10
0
Group
DW4
DW0
CW4
CW0
BW4
BW0
AW4
AW0
g__Lactobacillus
g__HT002
g__Romboutsia
g__Ignavigranum
g__Bifidobacterium
g__Corynebacterium
g__Facklamia
g__Candidatus_Arthromitus
g__Jeotgalicoccus
g__Lysobacter
g__Eubacterium]...uminantium_group
g__Anaeroplasma
g__Flaviflexus
g__Atopostipes
g__Lactococcus
g__Brachybacterium
g__Methylobacte...um−Methylorubrum
g__Aerococcus
g__Jeotgalibaca
g__Hydrogenophaga
g__Alloiococcus
g__Alcaligenes
g__Enteractinococcus
g__Paenalcaligenes
g__Phascolarctobacterium
g__Arthrobacter
g__Dietzia
g__Brevibacterium
g__Odoribacter
g__Leucobacter
